# CD44v6-mediated regulation of gastric cancer stem cells: a potential therapeutic target

**DOI:** 10.1007/s10238-025-01611-4

**Published:** 2025-03-12

**Authors:** Hao Chen, Ruoyu Ling, Jiayu Lai, Zhiqi Liu, Zhe Wang, Hua Yang, Yi Kong

**Affiliations:** 1 Department of General Surgery and Guangdong Provincial Key Laboratory of Precision Medicine for Gastrointestinal Tumor, Nanfang Hospital, Southern Medical University, Guangzhou, 510515 Guangdong Province China; 2 The First School of Clinical Medicine, Southern Medical University, Guangzhou, 510515 Guangdong Province China; 3 Department of Oncology, The First Affiliated Hospital of Guangdong Pharmaceutical University, Guangzhou, 510080 Guangdong Province China; 4 Department of Basic Medicine, School of Medicine, Foshan University, Foshan, 528225 Guangdong Province China; 5 Faculty of Pharmaceutical Sciences, Shenzhen University of Advanced Technology (SUAT), Shenzhen, 518107 Guangdong Province China

**Keywords:** Gastric cancer, CD44v6, Cancer stem cell, Tumor progression, Chemoresistance

## Abstract

Gastric cancer is the fourth most common cause of cancer-related deaths globally. Cancer stem cells (CSCs) play an essential role in tumor initiation, development, and chemoresistance. However, the molecular mechanisms that regulate CSC traits in gastric cancer, particularly the role of CD44v6 as a key CSC marker, remain poorly understood. Here, we demonstrate that CD44v6 is markedly upregulated in gastric cancer tissues and correlates with poor prognosis. Functional assays, including colony formation, wound healing, proliferation, and apoptosis assays, show that CD44v6 enhances CSC characteristics, such as self-renewal, proliferation, migration, and cisplatin chemoresistance. CD44v6 knockdown effectively suppresses these aggressive phenotypes. Mechanistically, CD44v6 regulates the expression of key CSC markers, including CD24, CD133, EpCAM, as well as stemness-related transcription factors Oct-4 and Nanog. Additionally, CD44v6 enhances cell proliferation and drug resistance in both in vitro and in vivo experiments. Collectively, our findings highlight the significant role of CD44v6 in regulating gastric CSC traits, suggesting it’s a potential as a biomarker and therapeutic target for improving gastric cancer treatment outcomes, particularly in overcoming chemoresistance.

**Supplementary Information:**

The online version contains supplementary material available at 10.1007/s10238-025-01611-4.

## Introduction

Gastric cancer ranks as the fourth most frequent cause of cancer-related death globally [1]. In spite of improvements in radical gastrectomy and the use of adjuvant radiotherapy and chemotherapy, postoperative recurrence rates range from 30 to 40%, and the overall prognosis for patients with gastric cancer remains poor [2,3]. These therapeutic challenges highlight the urgent need to identify specific tumor markers and therapeutic targets to improve patient outcomes.

Over the past decades, scientists have uncovered the essential role that cancer stem cells (CSCs) in tumor initiation, development, recurrence, as well as resistance to therapy [4–7]. CSCs, referred to as tumor-initiating cells, are a small subset of cancer cells possessing unique traits such as self-renewal, high differentiation potential, and drug resistance [8,9]. These properties enable CSCs to evade conventional chemotherapy and radiation, remaining dormant during treatment and later regenerating to repopulate the tumor [10,11]. Residual CSCs are thus considered the main culprits of tumor recurrence, metastasis, and treatment failure [12,13]. Identifying effective target molecules within CSCs is crucial for developing therapeutic strategies to improve patient outcomes. However, in gastric cancer, the mechanisms underlying CSC behavior remain poorly understood.

Identifying and isolating CSC phenotypes is crucial for targeted therapy research. CD44, CD24, Prominent-1 (CD133), and EpCAM are selectively expressed or elevated in CSCs, and are directly associated with tumor recurrence [10, 14–16]. Among these markers, CD44 is expressed as a standard form (CD44s) and also in multiple variants (CD44v) created by alternative mRNA splicing [17]. Specifically, CD44 variant 6 (CD44v6) has been implicated in carcinogenesis, tumor development, recurrence, and poor prognosis across multiple cancers, including colorectal and pancreatic cancers, where it serves as a marker of CSCs [18–21]. These findings indicate that CD44v6 might be significant in the progression of gastric cancer and requires further investigation as a possible therapeutic target.

This study attempts to explore the role of CD44v6 in regulating CSC traits, including tumor growth, proliferation, and resistance to apoptosis in gastric cancer, through in vivo and in vitro experiments. Moreover, we sought to identify CD44v6 as a biomarker for gastric CSCs and investigate its potential as a therapeutic candidate in clinical settings.

## Materials and method

### Data retrieval, extraction, and analysis of the cancer genome atlas program and gene expression omnibus data sets

The Cancer Genome Atlas Program (TCGA) database was searched using gastric cancer as the keyword to find the most significant gene expression difference between tumor tissue and normal tissue, The differential gene expression (DEG) was categorized into high and low expression group to further evaluate its impact on patient survival and prognosis. Finally, the DEGs were analyzed to find the most relevant pathways. Next, transcriptomic data from 33 common tumors in the TCGA-STAD dataset were processed with the One Class Linear Regression (OCLR), a machine learning algorithm focused on single-class logistic regression. Accordingly, the dryness of tumor samples was quantified and the mRNAsi (indicating the gene expression traits of stem cells) and EREG-mRNAsi (representing the epigenetically regulated stemness index derived from DNA methylation and transcriptomic data) of each sample were calculated using One Class Linear Regression. Spearman correlation analysis was applied to evaluate the relationship between differential genes and stem cell stemness in the Gene Expression Omnibus (GEO) data set (GSE83881).

### In vitro experiment

#### Cell culture

Human gastric cancer cell lines MKN-45 and NCI-N87 were sourced from the Cell Bank of Type Culture Collection of the Chinese Academy of Sciences in Shanghai, China. Each cell line was grown in RPMI-1640 medium with 10% fetal bovine serum (FBS), 100 U/ml penicillin, and 100 µg/ml streptomycin. The cells were cultured in a humidified atmosphere containing 5% CO_2_ at 37 °C. Once the gastric cancer cells were stained with trypan blue, they were examined for cell viability using light microscopy. A cell viability level greater than 95% was considered adequate.

#### Transfection and cloning of cells

CD44v6 shRNA was cloned into a pLVX-shRNA2-Puro-vector (Shanghai Genechem Co., Ltd.). The resulting pLVX-shRNA2-Puro-CD44v6 shRNA (CD44v6 shRNA) transfectants constitutively silence CD44v6 genes in MKN-45 and NCI-N87 cell lines (henceforth referred to as CD44v6-cells). To control the CD44v6 shRNA transfection, pSicoR-scrambled shRNA transfectants were employed. The v6 isoform of CD44, referred to as CD44v6, was expressed in the MKN-45 and NCI-N87 cell lines through transfection with the CD44-04-ENST00000415148 variant from OriGene. This variant was cloned into a pCDH-CMV-MCS-EF1-copGFP-T2A-Puro expression vector obtained from Shanghai Genechem Co., Ltd. The pCDH-CMV-MCS-EF1-copGFP-T2A-Puro empty vector was utilized as the control. The transfection process utilized Lipofectamine 2000 (Invitrogen, USA). To select clones stably CD44v6 + and CD44v6−, cells after transfection were exposed to puromycin (Beyotime Biotechnology, ST551, China) for 2 weeks. Transfection and selection efficiency were determined through real-time quantitative PCR (qRT-PCR) and Western blotting.

#### Real-time PCR

RNA was extracted from the samples using the TRIZOL® Reagent (Invitrogen, USA). TaKaRa's PrimeScriptTM 1st Strand cDNA Synthesis Kit was employed to synthesise the first strand of cDNA. TaKaRa's SYBR® Green PCR kit was utilized to perform the real-time PCR (RT-PCR). Table S1 in the Supplementary Materials shows the primers.

#### Soft agar and colony formation assay

The cells were analyzed for anchorage-independent growth in 6-well plates in triplicate. A 1% agarose solution, 0.6% agarose solution, and 2X RPMI-1640 medium/20% FCS (containing antibiotic) were kept at 40 °C in a water bath. For the base layer, the 1% agarose solution was 1:1 diluted with 2X RPMI-1640 medium containing 20% FCS, achieving a 0.5% agarose concentration, and 2 ml of the mixture solution was layered in each well. After allowing the base layer of agarose to set for 30–60 min at room temperature, top layer was added. The cells were suspended in 0.6% agarose solution which was 1:1 diluted with 2X RPMI-1640 medium containing 20% FCS to get a final ultimate of 0.3% agarose. A total volume of 2 ml, containing 1000 cells per well, was seeded. The agarose plate was placed in the incubator at 37 °C. After 2–3 weeks, colony formation was monitored under a phase-contrast microscope and colonies were counted. The efficiency of colony formation (%) = number of colonies / number of plated cells × 100.

#### Cell viability assay

In accordance with the instructions, cell proliferation capacity was detected using the Cell Counting Kit 8 (CCK-8) assay (Beyotime, China). A total of 1,000 cells were placed in each well of a 96-well plate and left to grow for the specified times. After 2 h, 10 μL of CCK-8 reagent was added to each plate at 37 °C and incubate for one more hour. Optical density readings were taken at 450 nm, and each experiment was conducted in three separate trials.

#### Apoptosis assay

Propidium iodide (PI) and AnnexinV-FITC were used in a double staining procedure to identify necrotic and apoptotic cells. To assess cellular apoptosis, 1 × 10 [5] cells were seeded into a 96-well plate and exposed to 50 μg of cisplatin for 48 h. Subsequently, cells were rinsed with PBS containing 1% FCS. In order to evaluate cell survival, the cells were treated with AnnexinV-FITC/PI, according to the instructions, and kept in the dark for 15 min. The apoptotic status of cells was evaluated by flow cytometry with two channels.

#### Wound-healing assay

Cells were grown in a 6-well plate until approximately 90% confluence was achieved, under standard conditions (37 °C, 5% CO₂). A uniform wound was created in the center of each well by scratching the monolayer with a sterile 200-µL pipette tip. Wound closure was assessed using light microscopy 48 h after the procedure. The percentage of wound closure was measured using ImageJ software by calculating the reduction in wound area over time.

#### Western blotting

MKN-45 and NCI-N87 cells were seeded at a density of 5 × 10 [5] cells per dish in 60-mm culture dishes and left to incubate for 24 h. Western blotting was as previously described [22]. A quantity of 20 μg of protein was mixed with sample buffer and separated by 10% SDS–polyacrylamide gel electrophoresis. The resolved proteins were transferred to a PVDF membrane. Subsequently, the membrane was blocked with 5% skimmed milk in Tris-buffered saline with 0.1% Tween® 20 detergent (TBST). The membranes were incubated overnight with primary antibodies at a temperature of 4 °C. The membranes were exposed to a peroxidase-conjugated secondary antibody (all obtained from Cell Signalling Technology, USA) at a 1:2000 dilution during the incubation process. The ECL Western Blotting Detection System (GE Healthcare, UK) was employed to identify signals, which were then captured on X-ray film (Fuji Photo Film, Japan). The primary antibodies are listed in Table S2.

### Animal experiments

#### Animal model

Five-week-old female BALB/c nude mice were acquired from Animal Research Center of Southern Medical University in Guangzhou, China. All experiments involving animals were approved by the Nanfang Hospital Ethics Committee for animal experiments. Each mouse received a subcutaneous injection of MKN-45 gastric cancer cells (1 × 10 [6]/100 μL PBS) in the dorsal flank. Six mice were randomly separated into two groups (n = 3 per group): the CD44v6 knockdown group and the control group. A caliper was utilized to measure the length (L) and width (W) of the tumor every three days in order to track its size, and the volume (V) of the tumor was determined using the formula: V = (L × W [2])/2. We weighed the tumors and euthanized the mice twenty days after injection.

In the experimental process, we strictly followed randomization and blinding protocols. The animals were randomly divided into two groups to minimize potential confounding factors. Both the operators and evaluators were blinded throughout the study to minimize subjective bias, thereby enhancing the objectivity and reliability of the study results.

#### Immunohistochemistry

The immunohistochemistry (IHC) procedure was conducted using the PV-9000 kit (ZSGB-BIO, China) following the manufacturer’s instructions. Paraffin-embedded tissue sections were subjected to immunohistochemical staining using primary antibodies against CD44 (1:500, Proteintech, 15675-1-AP, China), CD44s (1:50, R&D Systems, MAB7045), CD44v3 (1:100, Bioss, bs-4497R, China), CD44v4 (1:100, Bioss, bs-2783R, China), CD44v5 (1:100, Bioss, bs-2782R, China), CD44v6 (1:50, Abcam, ab254174, USA), CD44v7-8 (1:30, Invitrogen, BMS118, USA), CD44v9 (1:100, BioLegend, 394402, USA), and Ki67 (1:100, Invitrogen, 14-5698-82, USA), and incubated overnight incubation at 4 °C. After washing with PBS, the samples were treated with secondary antibodies conjugated with horseradish peroxidase for mice or rabbits for one hour at room temperature. Ultimately, the paraffin sections were mounted with coverslips and examined under a ZEISS microscopic (Japan).

#### Immunofluorescence

Immunofluorescence analysis was conducted on tissue microarrays. Tissue slides were deparaffinized in xylene and rehydrated through a graded ethanol series. To retrieve epitopes using heat, slides were microwaved for 20 min in DAKO Target Retrieval solution (pH 6). Following the blocking step, slides were incubated at 4 °C overnight with primary antibodies: CD44v6 (1:50, Abcam, ab254174, USA), CD133 (1:50, Invitrogen, PA5-38014, USA), and CD24 (1:50, Invitrogen, MA5-11828, USA). Secondary antibody incubation was performed for one hour at room temperature. Slides were mounted with DAPI, and the images were captured by a ZEISS microscope from Japan.

### Statistical analysis

Each study was conducted with a minimum of three repetitions. The data for the figure are presented as the mean value as mean ± standard error of the mean (SEM). The statistical analysis was performed using SPSS 25.0 software (SPSS Inc., USA). Student's t-test was utilized to compare the data obtained from two distinct groups. The differences in data among three or more groups were assessed using either repeated measures ANOVA or One-way ANOVA. A *P value* < 0.05 was considered statistically significant.

## Results

### Correlation analysis of CD44 and gastric cancer progression.

**Fig. 1:**
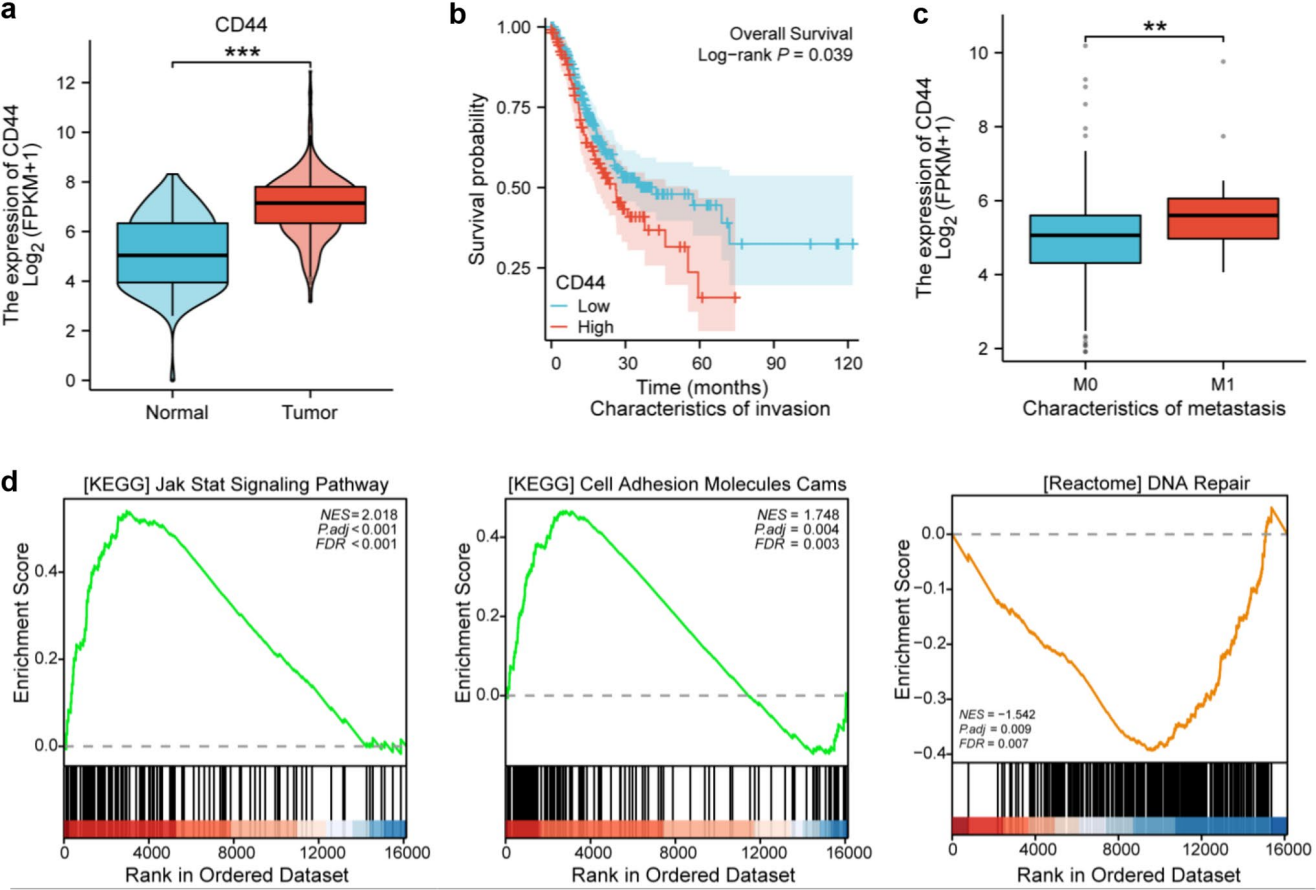
Correlation analysis of CD44 and gastric cancer progression. **a** Through the analysis of TCGA-STAD (Gastric cancer) dataset, it was found that the expression of CD44 in tumor tissues was higher than that in normal tissues. **b** Survival analysis showed that the prognosis of the group with high CD44 expression was worse than that of the group with low CD44 expression. **c** The expression of CD44 in patients with metastasis was higher than that in patients without metastasis. **d** Enrichment analysis of differentially expressed genes in CD44 high expression and CD44 low expression groups

To clarify the function of CD44 in gastric cancer, we analyzed the TCGA-STAD dataset and observed that the expression levels of CD44 in gastric cancer tissues were significantly elevated compared to those in normal tissues (*p* < 0.001, Fig. 1a). Based on the expression levels of CD44, the tumor samples were divided into two groups: one group exhibited high CD44 expression, while the other exhibited low CD44 expression. Our analysis revealed that high CD44 expression was linked to a poorer prognosis compared to the group with low CD44 expression (*p* = 0.039, Fig. 1b). Similarly, CD44 expression level was markedly higher in patients diagnosed with M1 (metastatic disease) compared to those diagnosed with M0 (non-metastatic disease) (*p* < 0.01, Fig. 1c). The results of pathway enrichment analysis of differentially expressed genes showed that individuals with high CD44 expression levels are mainly linked to the JAK-STAT and cell adhesion pathways (green, NES > 1), whereas those with low CD44 expression are primarily enriched in the DNA repair pathway (orange, NES < 1) (Fig. 1d).

Clinically, the above results suggest a potential link between high CD44 expression and the advancement of gastric cancer. Given the significant role of CD44 expression in gastric cancer progression, it is crucial to further explore its relationship with other oncogenic properties, such as tumor stemness, to understand its broader impact on tumor biology.

### CD44v6 expression positively correlates with CSC marker levels in gastric cancer tissues

Based on bioinformatics data analysis, CD44 overexpression was correlated with poor prognosis and metastatic disease in gastric cancer. To further explore CD44 expression and the specific roles of its isoforms in gastric cancer, we examined clinical gastric cancer samples.

**Fig. 2:**
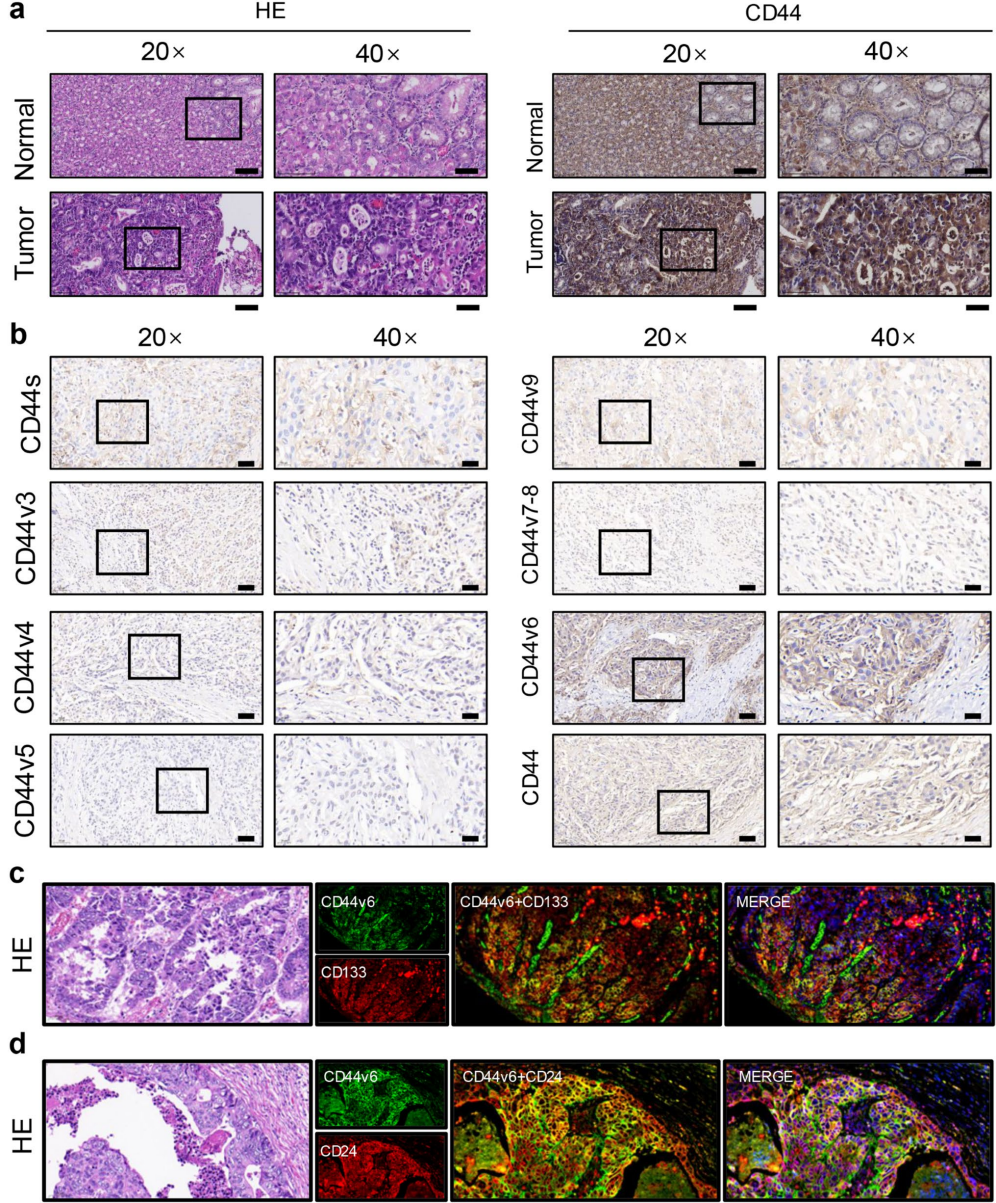
CD44v6 expression positively correlates with CSC marker levels in gastric cancer tissues. **a** Compared with normal tissues, the expression of CD44 was increased in gastric cancer, and the expression of its isomer CD44 was more significant. **b** Expression of CD44 isomers in gastric cancer. **c** CD133 and CD44v6 were co-expressed in gastric cancer. **d** CD24 and CD44v6 were co-expressed in gastric cancer

Hematoxylin–eosin (HE) staining revealed significant differences in tissue architecture between normal and tumor tissues. Furthermore, IHC analysis indicated a notable increase in CD44 expression in gastric cancer tissues relative to adjacent normal tissues, underscoring the potential role of CD44 in promoting tumor progression (Fig. 2a).

The overall expression of CD44 may mask the distinct functions of its isoforms. To further investigate the roles of CD44 isoforms in gastric cancer development, we performed additional IHC analysis. Among CD44 isoforms, CD44v6 exhibited the highest expression in gastric cancer tissues, significantly surpassing other isoforms, including CD44s, CD44v3-v5, and CD44v7-v9. CD44v6 expression was predominantly localized to the cell membrane, consistent with its proposed function as a CSC surface marker (Fig. 2b).

Building on the reported association between CD44v6 and tumor stemness in other cancers [23], we sought to further investigate its specific role in gastric CSCs. CD133 and CD24, recognized as hallmarks of CSCs, were examined using immunofluorescence staining. Regions with elevated CD44v6 expression colocalized with increased levels of CD133 (Fig. 2c) and CD24 (Fig. 2d). Collectively, these results show that CD44v6 is crucial for maintaining stem cell-like properties in gastric cancer cells and in driving gastric cancer development, particularly through its association with CSC traits.

### CD44v6 modulates CSC marker expression and maintains gastric CSC characteristics

Following our findings of significantly increased CD44v6 expression in gastric cancer tissues and its correlation with CSC markers, we next investigated its functional role by examining how CD44v6 expression levels affect gastric CSC characteristics and related mechanisms.

**Fig. 3:**
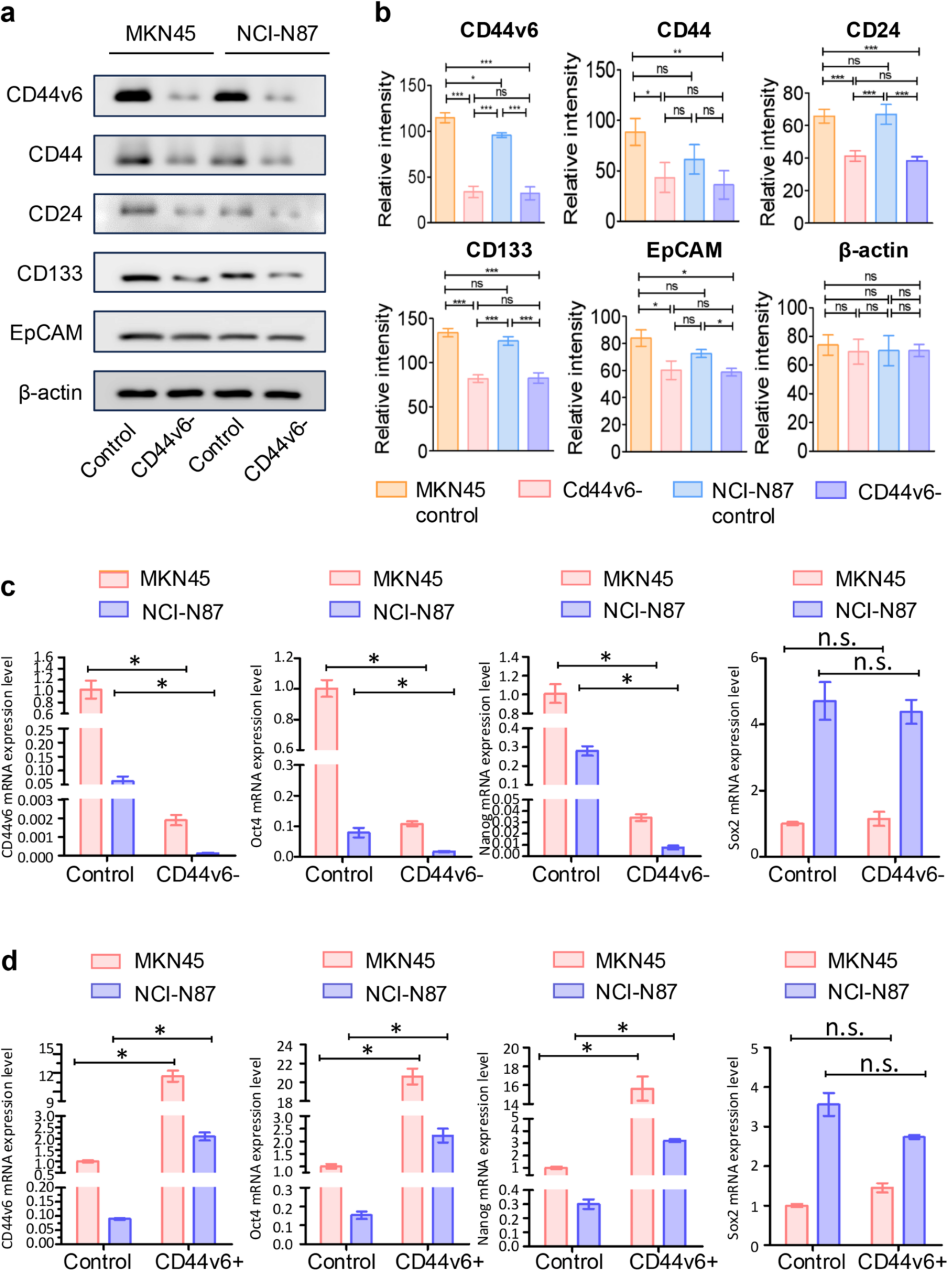
CD44v6 modulates CSC marker expression and maintains gastric CSC characteristics. **a**, **b** CD44v6^−^ in MKN-45 and NCI-N87 cells was controlled by Western blot to evaluate the CSC markers CD44, CD24, CD133, and EpCAM. **c**, **d** Both CD44v6^−^ and CD44v6^+^ in MKN-45 and NCI-N87 cells were performed by real-time PCR to evaluate the CSC-related genes Oct4, Nanog, and Sox2, respectively. Significant differences to control cells: * *p* < 0.05, **p < 0.01, and ***p < 0.001

Specifically, two gastric cancer cell lines, MKN-45 and NCI-N87, were utilized as experimental models to investigate the effects of CD44v6 knockdown and overexpression. Western blot analyses confirmed effective CD44v6 knockdown in both cell lines, showing significantly reduced relative intensity compared to the control group (*p* < 0.05, Fig. 3a). The reduction in CD44v6 was accompanied by decreased levels of CD44, CD24, and CD133, which are widely recognized as key CSC markers involved in regulating self-renewal, differentiation potential, and tumor aggressiveness (*p* < 0.001). Additionally, EpCAM expression, a molecule critical for cell adhesion and CSC maintenance, showed a moderate to weak decrease (*p* < 0.05, Fig. 3b).

Furthermore, the knockdown of CD44v6 resulted in a significant decrease in the mRNA levels of Oct-4 and Nanog, two vital transcription factors for sustaining self-renewal and pluripotency (*p* < 0.05). However, Sox-2, another transcription factor, remained unaffected by CD44v6 modulation (Fig. 3c). Conversely, overexpression of CD44v6 led to increased levels of Oct-4 and Nanog, with Sox-2 expression remaining unchanged (Fig. 3d).

These observations demonstrate that CD44v6 plays a pivotal role in regulating key CSC markers and maintaining the stem cell-like properties of gastric cancer cells.

### CD44v6 enhances self-renewal, proliferation, and migration in gastric cancer cells

Building on the role of CD44v6 in regulating CSC marker expression, we further assessed its impact on the self-renewal, colony formation, migration, and proliferation abilities of gastric cancer cells.

**Fig. 4:**
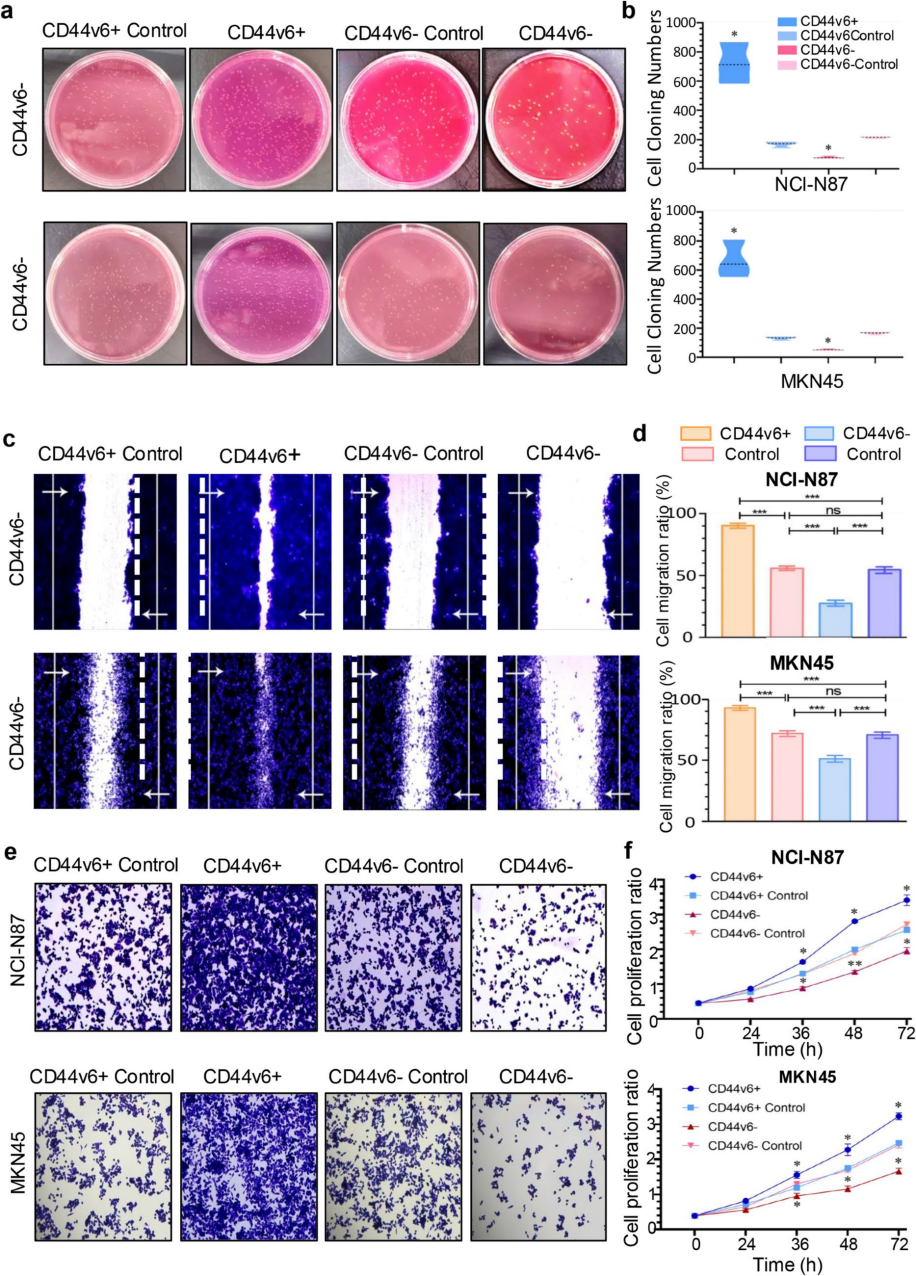
CD44v6 enhances self-renewal, proliferation, and migration in gastric cancer cells. **a** Anchorage-independent growth capacity of both CD44v6^−^ and CD44v6^+^ in MKN-45 and NCI-N87 cells were characterized by soft agar colony formation assays. **b** Quantitative analysis of colony numbers and size in soft agar assays. **c** Subconfluent monolayers of cells as in were scratched with a pipette tip. Wound healing was recorded for 48 h. **d** Quantitative analysis of scratch test. **e** Both CD44v6^−^ and CD44v6^+^ in MKN-45 and NCI-N87 cells were controlled for cell proliferation by crystal violet and CCK-8. **f** Quantitative analysis of cell proliferation. A representative example of CD44v6^−^, CD44v6^+^ and control cells and mean ± SD of the wound area in three independent assays are shown. Mean ± SD of three independent assays are shown, significant differences to control cells: * *p* < 0.05, **p < 0.01, and ***p < 0.001

The colony formation assay showed that MKN-45 ^CD44v6+^ and NCI-N87 ^CD44v6+^ cells exhibited significantly enhanced colony formation (p < 0.05), while MKN-45 ^CD44v6−^ and NCI-N87 ^CD44v6−^ cells showed markedly reduced colony formation than the control cells (*p* < 0.05), indicating a pivotal role of CD44v6 in supporting self-renewal characteristics (Fig. 4a, b). Microscopic examination of the soft agar colony formation assay showed that CD44v6− cells formed fewer and smaller colonies in comparison with the control group (Fig. S1a). Quantitative analysis confirmed a marked decrease in the number of cell clusters formed by CD44v6− cells (*p* < 0.001, Fig. S1b). Additionally, the colony diameter of CD44v6− cells was significantly smaller than that of the control group (*p* < 0.001, Fig. S1c, S1d). This evidence underscores the critical function of CD44v6 in enhancing the self-renewal and tumorigenic capabilities of gastric cancer cells, thereby supporting its potential as a therapeutic intervention point in gastric cancer.

The migration of CD44v6^−^, CD44v6^+^, and control cells was assessed using the wound healing assay. Notably, the wound healing rate was significantly enhanced in MKN-45^CD44v6^^+^ and NCI-N87^CD44v6+^ cells, while CD44v6− cells exhibited weaker migration abilities than the control group. (*p* < 0.001, Fig. 4c, d).

Additionally, the viability of CD44v6^−^, CD44v6^+^, and control cells was assessed using crystal violet and CCK-8 assays. Cell viability, assessed using crystal violet staining, showed that CD44v6 + cells had a significantly increased survival rate compared to both control and CD44v6− cells in both gastric cancer cell lines (Fig. 4e). The proliferation of CD44v6^−^ cells was notably diminished at 36 h, 48 h, and 72 h (*p* < 0.05). In contrast, CD44v6^+^ cells exhibited heightened proliferation compared to control cells, particularly relative to CD44v6− cells (*p* < 0.05, Fig. 4f).

Overall, these results establish CD44v6 as a key enhancer of multiple malignancy-associated behaviors in gastric cancer cells, underscoring its importance as a promising therapeutic intervention to inhibit gastric cancer progression.

### CD44v6 enhances chemotherapy resistance in gastric cancer cells

**Fig. 5:**
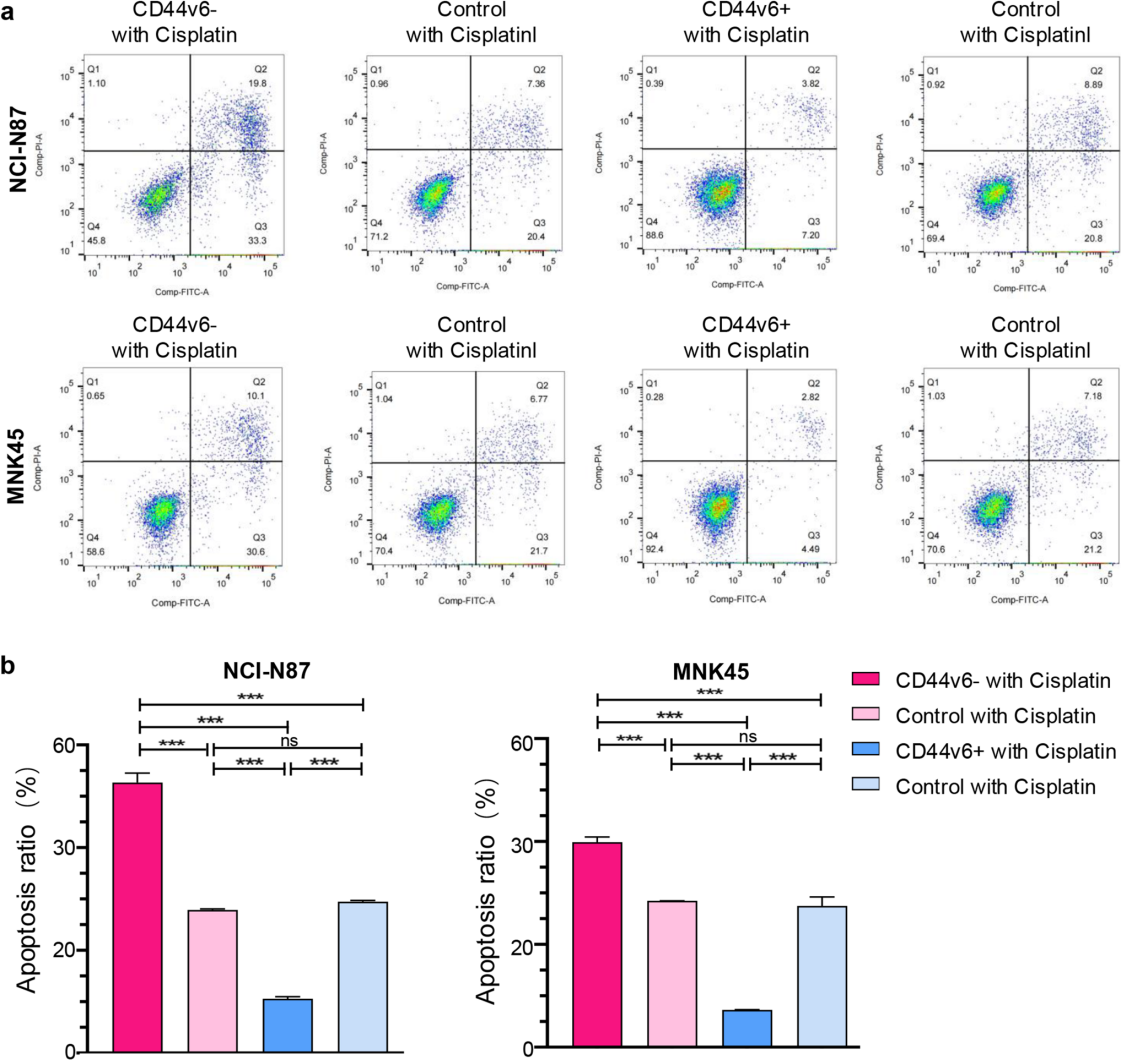
CD44v6 enhances chemotherapy resistance in gastric cancer cells. **a**, **b** Apoptosis was evaluated by Annexin V/PI staining after 48-h culture in identical amounts of cisplatin (50 μg/ml). Mean ± SD of three independent assays are shown, significant differences to control cells: * *p* < 0.05, **p < 0.01, and ***p < 0.001

After demonstrating the notable effect of CD44v6 on self-renewal, colony formation, proliferation, and migration of gastric cancer cells, we next explored its potential impact on chemotherapy resistance, specifically its effect on apoptosis triggered by cisplatin treatment. Cells were treated with 50 μg/mL cisplatin for a duration of 48 h, and apoptosis was assessed via Annexin V/PI staining. The results confirmed that the cisplatin administration effectively decreased the apoptosis resistance of CD44v6^−^ cells compared to control cells (*p* < 0.001). By contrast, CD44v6^+^ cells exhibited significantly enhanced apoptosis resistance in both MKN-45 and NCI-N87 cell lines when compared to both CD44v6^−^ and control cells (*p* < 0.001), as seen in Fig. 5a, b.

CD44v6 not only influences the biological behaviors of gastric cancer cells but also significantly impacts their response to the chemotherapeutic cisplatin. Our data underscore the importance of CD44v6 as a prospective treatment target, especially in designing strategies to combat drug resistance.

### CD44v6 knockdown suppresses subcutaneous tumor growth and stem cell marker expression in gastric cancer models

**Fig. 6:**
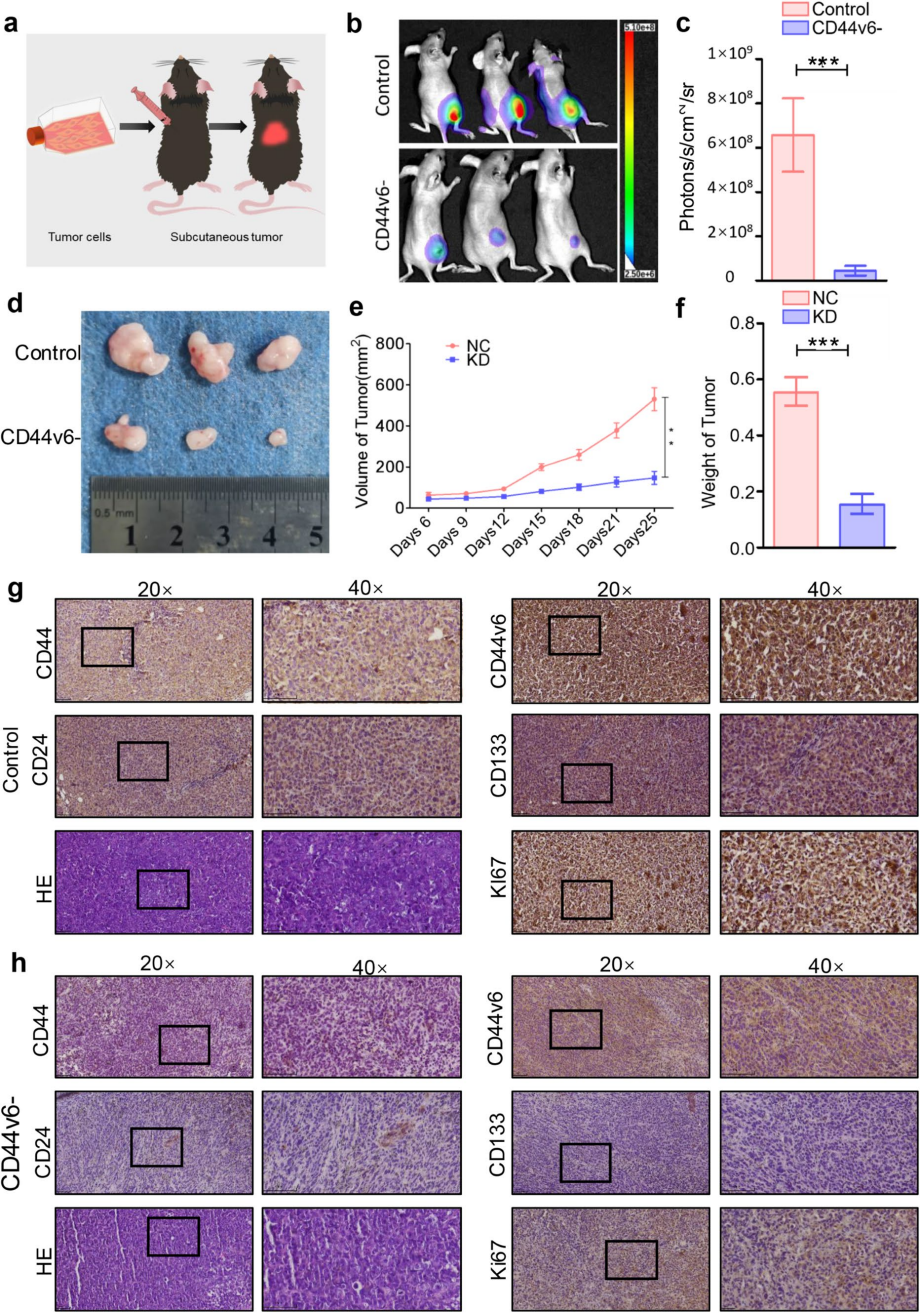
CD44v6 knockdown suppresses subcutaneous tumor growth and stem cell marker expression in gastric cancer models. **a** Animal subcutaneous tumor modeling diagram. **b** Control group and CD44v6 knockdown group were imaged in vivo. **c** Fluorescence quantitative analysis in vivo imaging of animals. **d**, **e** Gross and quantitative analysis of tumor volume in experimental group and control group. **f** Quantitative analysis of tumor weight in experimental group and control group. **g** HE staining and expression of CD44, CD44v6, CD24, CD133 and Ki67 in the control group. **h** HE staining and expression of CD44, CD44v6, CD24, CD133, and Ki67 in the CD44v6 knockdown group. Mean ± SD of three independent assays are shown, significant differences to control cells: * *p* < 0.05, **p < 0.01, and ***p < 0.001

To verify the regulatory impact of CD44v6 on gastric cancer, tumor growth was monitored in both the control and CD44v6 knockdown groups using in vivo imaging techniques. The experimental process is shown in Fig. 6a. Results indicated that tumor growth was significantly suppressed in the knockdown group than the control group, with a notable reduction in fluorescence intensity (*p* < 0.001, Fig. 6b, c). Macroscopically, both the appearance and size of tumors were significantly reduced in the CD44v6 knockdown group, further verifying the suppressive impact of gene knockdown on tumor growth (Fig. 6d). Detailed measurement data showed that, when compared to the control group, CD44v6 knockdown significantly reduced tumor volume (*p* < 0.01, Fig. 6e) and weight (*p* < 0.001, Fig. 6f).

Histological examination revealed that in the control group, HE staining and the expression of CD44, CD44v6, CD24, CD133, and Ki67 were markedly highly expressed (Fig. 6g). In contrast, in the CD44v6 knockdown group, both HE staining and the expression of these tumor markers were significantly reduced, closely correlating with the slower tumor growth (Fig. 6h).

**Fig. 7:**
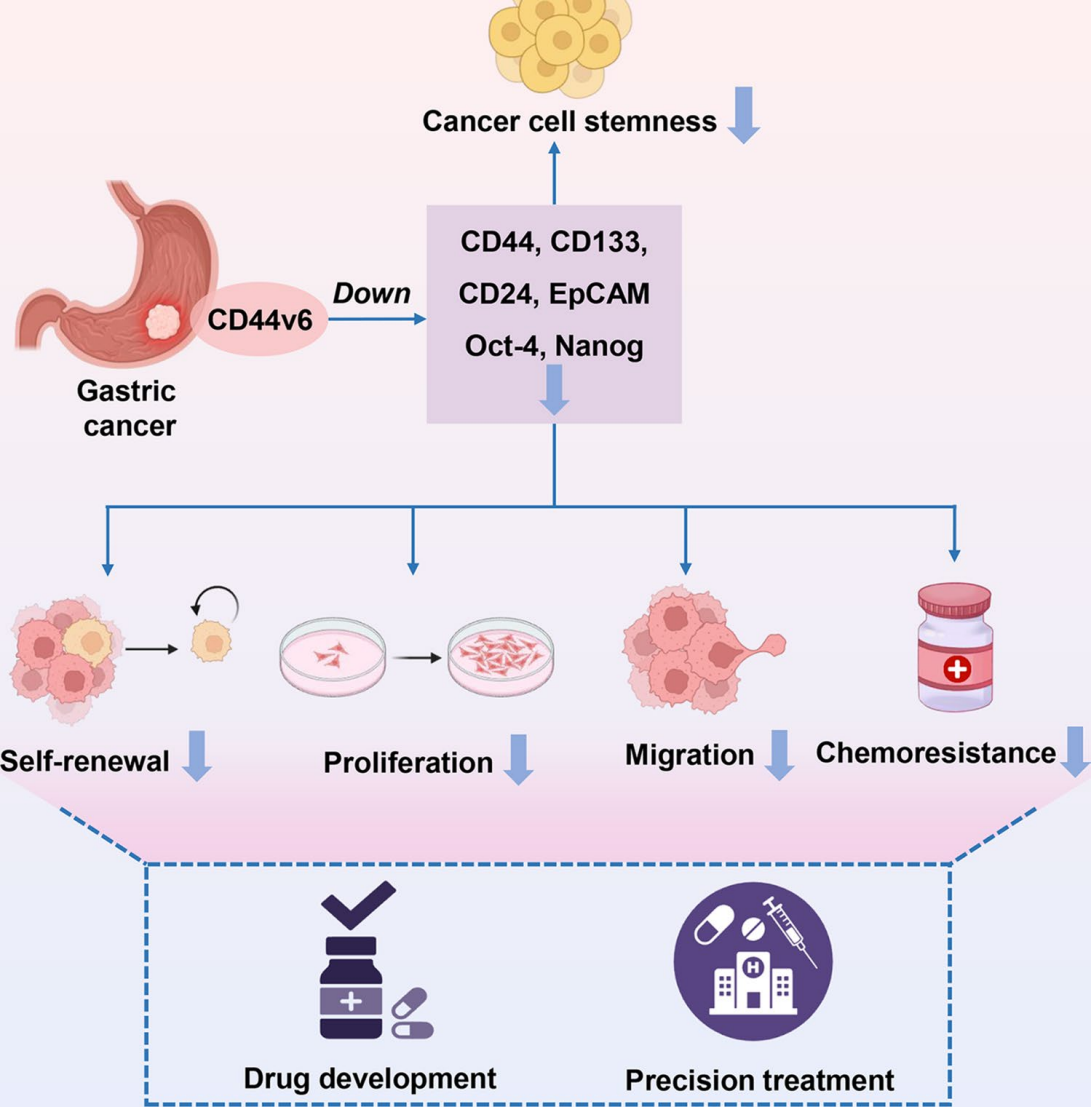
Schematic diagram of targeting CD44v6 to inhibit gastric cancer progression as a potential therapy

Collectively, these in vivo findings highlight the crucial regulatory role of CD44v6 in gastric cancer progression, emphasizing its promise as a target for precision therapy and drug development (Fig. 7).

## Discussion

In this research, we highlight the pivotal role of CD44v6 in gastric cancer progression, particularly its connection to CSC traits. CD44v6 was markedly upregulated in gastric cancer tissues, correlating with a worse prognosis and enhancing CSC traits by increasing the expression of key markers such as CD44, CD133, and EpCAM, as well as key transcription factors like Oct-4 and Nanog. Additionally, CD44v6 promoted malignant behaviors, including self-renewal, colony formation, proliferation, and migration, and contributed to chemoresistance through increased resistance to cisplatin. In vivo, CD44v6 knockdown effectively suppressed tumor growth and reduced CSC marker expression, highlighting its potential as a treatment target in gastric cancer. To the best of our knowledge, this is the first study to directly establish the functional role of CD44v6 in regulating CSC phenotypes and malignant behaviors in gastric cancer.

CD44v6, a well-recognized CSC surface marker [17], is involved in cancer metastasis, apoptosis resistance and tumorigenesis across multiple cancer types [20,21,24]. In gastrointestinal cancers, including colorectal, pancreatic [22], and gastric cancers [25,26], CD44v6 high expression has been strongly associated with poor prognosis. Studies have demonstrated significant correlations between elevated CD44v6 expression and worse clinical outcomes, including lymph node metastasis, advanced TNM stages, and reduced survival in gastric cancer patients [25,26]. However, these studies primarily focused on the expression levels of CD44v6, leaving its role as a functional regulator of CSC traits unclear. Given the high heterogeneity, aggressive nature, and elevated recurrence rates of gastric cancer, understanding the unique mechanisms of stemness regulation by CD44v6 is critically important [27]. CSCs are considered key drivers of treatment failure and recurrence, making it essential to elucidate their regulatory pathways to develop more precise therapeutic strategies [28]. Our study represents the first functional investigation into how CD44v6 directly regulates CSC phenotypes and malignant behaviors in gastric cancer. This discovery not only provides new insights into the mechanisms underlying gastric cancer progression but also highlights the potential of CD44v6 to serve as a therapeutic target, laying the groundwork for future studies on its role in tumor development and treatment response.

In our study, through both in vitro and in vivo experiments, we confirmed that CD44v6 plays a crucial role in regulating CSC traits and promoting malignant behaviors in gastric cancer. Suppressing CD44v6 in gastric cancer cell lines resulted in a significant decrease in anchorage-independent growth, reduced colony formation capacity, and diminished migration and proliferation. Additionally, knockdown of CD44v6 sensitized gastric cancer cells to cisplatin-induced apoptosis, highlighting its role in chemoresistance. Conversely, CD44v6 overexpression restored these aggressive phenotypes, further substantiating its role as a functional regulator of CSC-related traits. These qualities align with the efforts of our organization and several others [22,29]. Importantly, our study provides a functional framework for utilizing CD44v6 as a candidate target in precision therapy for gastric cancer. The insights gained from these findings could inform the design of clinical trials aimed at testing CD44v6-targeted therapies, particularly in the context of overcoming chemoresistance and reducing tumor recurrence.

Mechanistically, CD44v6 influenced the expression of key CSC markers, including CD24, CD133, and EpCAM, which are known to be associated with cancer progression, stemness, and malignant behaviors like self-renewal, proliferation, migration, and chemoresistance [10,30–34]. In our study, we found that CD44v6 upregulates these markers, contributing to enhanced tumorigenic potential and increased resistance to cisplatin in gastric cancer cells. This regulatory effect highlights CD44v6’s pivotal role in maintaining the aggressive cancer cell phenotype and driving gastric cancer progression through its regulation of key CSC traits.

In addition to regulating CSC markers, CD44v6 also impacts stemness-related transcription factors like Oct-4 and Nanog, both of which are essential for self-renewal and pluripotency in CSCs [35]. Our results show that CD44v6 positively regulates the mRNA levels of Oct-4 and Nanog in gastric cancer cells, supporting their central role in maintaining stemness and promoting tumor progression. The findings align with the research conducted by Li et al., indicating that Oct-4 and Nanog could serve as valuable prognostic indicators for tumor relapse or metastasis [36]. Notably, Oct-4 demonstrated a stronger regulatory influence than Nanog, underscoring its central role in CSC biology within gastric cancer. Interestingly, Sox-2 expression remained unchanged regardless of CD44v6 manipulation, suggesting that Sox-2 operates through a distinct regulatory mechanism independent of CD44v6. The function of Sox-2 in gastric cancer is still being debated. While some studies associate Sox-2 overexpression with increased tumor aggressiveness and poor prognosis [37,38], others suggest the opposite [36]. This suggests that the regulation of Sox-2 may be independent of CD44v6 and potentially mediated through other signaling pathways. Sox-2 is key to promoting gastric cancer cell proliferation and chemoresistance [39], thus exploring its regulatory network may uncover mechanisms driving tumor aggressiveness, recurrence, and therapy resistance, offering new targets for treatment.

The relationship between CD44v6 and chemoresistance in gastric cancer remains controversial. Some studies have reported that CD44v6 knockdown can make gastric cancer cells resistant to platinum drugs [40,41], while others have suggested that gastric cancer patients with high CD44v6 expression should be selected for conventional chemotherapy in conjunction with surgery [27]. Notably, our study suggests that CD44v6 potentially influences cisplatin response through the regulation of stemness traits, particularly self-renewal and proliferation. This discrepancy may be due to variations in chemotherapy regimens, tumor staging, or research methodologies. It might also be linked to the role of CD44v6 in regulating key CSC functions such as self-renewal and proliferation [42]. Therefore, further studies are required to explore the core mechanisms through which CD44v6 induces chemotherapy resistance in gastric cancer. Previous reports suggest that CD44v6 influences chemotherapy resistance through the regulation of critical signaling pathways. As an illustration, CD44v6 has been shown to mediate chemoresistance in colorectal cancer by modulating the WNT/β-catenin signaling pathway and enhancing drug efflux [43]. Specifically, CD44v6 activates the WNT/β-catenin/TCF4 pathway to regulate CSC traits, thereby promoting drug resistance, including resistance to the FOLFOX regimen containing platinum-based drugs. In our study, we observed that elevated CD44 expression in gastric cancer tissues is related to significant enrichment of the JAK-STAT and cell adhesion pathways, both of which are known to play roles in cisplatin resistance [44,45]. Inhibition of the JAK/STAT pathway is known to promote apoptosis and reduce cisplatin resistance in gastric cancer [46], while the JAK/STAT pathway also supports CSC self-renewal, leading to chemoresistance [47]. Moreover, the cell adhesion pathway modulates gastric cancer cell sensitivity to cisplatin by regulating adhesion molecules such as Muc-1, ICAM-1, and VCAM-1 [45]. This pathway also contributes to the regulation of cell proliferation and apoptosis, further enhancing resistance. Additionally, as CSC chemoresistance is closely linked to their dormancy ^48^, whether CD44v6 affects CSC dormancy traits to drive chemoresistance remains to be investigated in future studies. Given the complex interactions between CD44v6, stemness traits, and chemoresistance, future research should aim to clarify the direct molecular mechanisms driving this process and explore the therapeutic potential of targeting CD44v6 in gastric cancer.

This study has several limitations. First, while we demonstrate the role of CD44v6 in regulating CSC traits and chemoresistance, these results are primarily based on observational data, and more direct mechanistic evidence would be valuable for strengthening this relationship. Second, the use of existing databases and specific cohorts may introduce selection bias, which could limit the generalizability of our findings to the broader gastric cancer population. Third, the sample size of the animal experiments is limited, and using an animal metastasis model potentially helps confirm the observations from the patient samples.

## Conclusions and future perspectives

Collectively, this study highlights the pivotal role of CD44v6 in regulating CSC traits, promoting malignant behaviors, and contributing to chemoresistance in gastric cancer. These findings significantly contributes to the understanding of gastric cancer pathology, particularly in the context of CSCs. In future, we will further elucidate the molecular mechanisms by which CD44v6 regulates CSC traits and chemoresistance, as well as explore the therapeutic potential of targeting CD44v6 to overcome chemoresistance. Moreover, we will expand the sample size in animal experiments and incorporate metastasis models to further validate our observations and gain a more comprehensive understanding of how CD44v6 contributes to gastric cancer progression.

By identifying CD44v6 as a critical biomarker and therapeutic target, our research sets the stage for future investigations into cancer stemness mechanisms and provides a foundation for improving treatment strategies for gastric cancer.

## Supplementary Information

Below is the link to the electronic supplementary material.

Supplementary file1 (PDF 2972 KB)

## Data Availability

The original contributions presented in the study are included in the article and supplementary materials. Further inquiries can be directed to the corresponding authors. Data is provided within the manuscript or supplementary information files.
