# Supplementary material for: CD44v6-mediated regulation of gastric cancer stem cells: a potential therapeutic target
Source: Clin Exp Med. 2025 Mar 12;25(1):80. doi: 10.1007/s10238-025-01611-4 (PMC11897096; doi:10.1007/s10238-025-01611-4)
Supplement: Supplementary file 1 — Supplementary file1 (PDF 2972 KB) [file 10238_2025_1611_MOESM1_ESM.pdf]

Supplementary data for

CD44v6-Mediated Regulation of Gastric Cancer Stem Cells: A Potential Therapeutic Target

Hao Chen [1#], Ruoyu Ling [1#], Jiayu Lai [2], Zhiqi Liu [2], Zhe Wang [3*], Hua Yang [4*], Yi Kong [5*]

1. Department of General Surgery & Guangdong Provincial Key Laboratory of Precision Medicine for Gastrointestinal Tumor, Nanfang Hospital, Southern Medical University, Guangzhou, China

2. The First School of Clinical Medicine, Southern Medical University, Guangzhou, China.

3. Department of Oncology, The First Affiliated Hospital of Guangdong Pharmaceutical University, Guangzhou, Guangdong Province, 510080, China

4. Department of Basic Medicine, School of Medicine, Foshan University, Foshan, Guangdong Province, 528000, China

5. Faculty of Pharmaceutical Sciences, Shenzhen University of Advanced Technology (SUAT), Shenzhen, Guangdong Province, 518107, China.

[#] These authors contributed equally to this work.

* Correspondence.

E-mail addresses: kongyi@suat-sz.edu.cn (Y, Kong), huayang@fosu.edu.cn (H, Yang), wangzhe@gdpu.edu.cn (Z, Wang)

Keywords: gastric cancer, CD44v6, cancer stem cell, tumor progression, chemoresistance

Supplementary figure

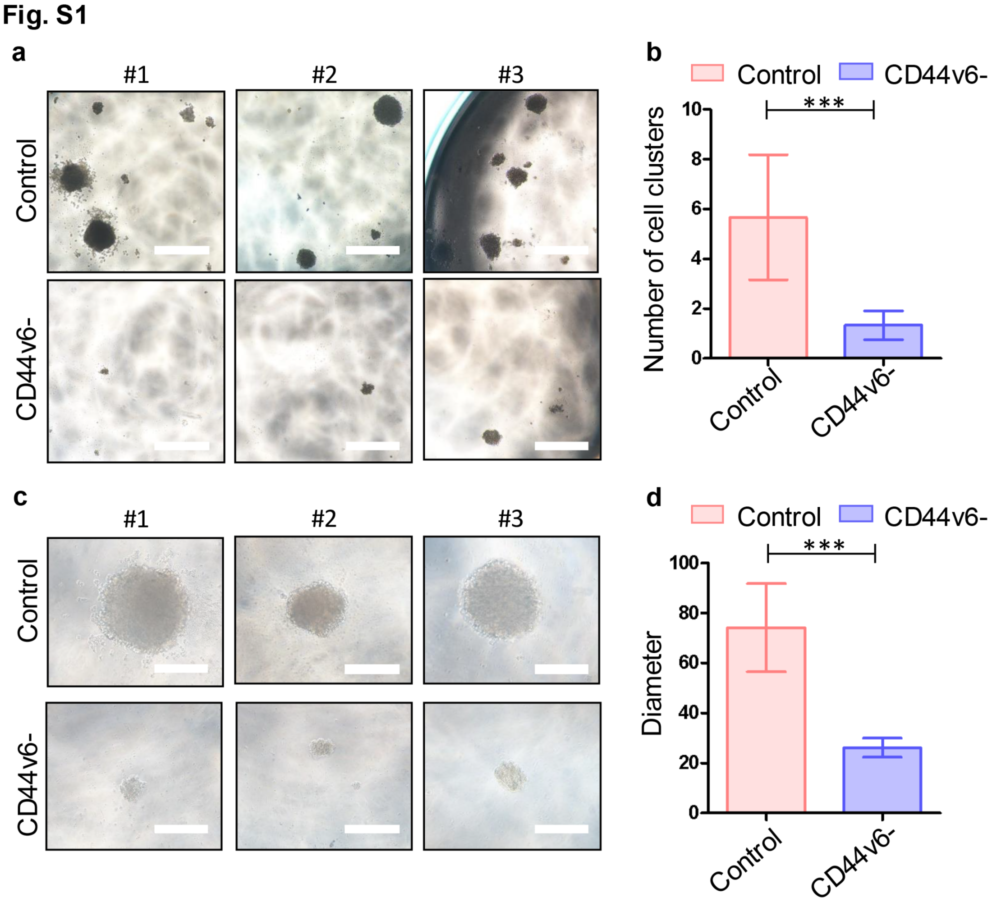

Fig.S1. Effect of CD44v6 knockdown on anchorage-independent growth of gastric cancer cells. (a) Soft agar colony formation assays demonstrate the anchorage-independent growth capacity of control and CD44v6 knockdown cells. Representative images show a reduced number and size of colonies in the CD44v6 knockdown group. Scale bars: 100 μm. (b) Quantitative analysis of the number of colonies formed shows a significant reduction in the CD44v6 knockdown group compared to the control group. (c) Microscopic examination highlights morphological differences between colonies formed by control and CD44v6 knockdown cells, with visibly smaller and less developed colonies in the knockdown group. Scale bars: 50 μm. (d) Quantitative analysis of colony diameter reveals significantly smaller colonies in the CD44v6 knockdown group compared to the control group. Mean±SD of three independent assays is shown, significant differences to control cells: * $p < 0.05$, ** $p < 0.01$, and *** $p < 0.001$.

Supplementary Tables

Table S1. Primer sequence used in this study.

Gene	sequence
**CD44V6**	
F	5′ GATGTCACAGGTGGAAGAAG 3′
R	5′ GGTCCTGCTTTCCTTCGTGT 3′
**Nanog**	
F	5′ AGAGAAGAGTGTCGCAAAAAAGGA 3′
R	5′ TGAGGTTCAGGATGTTGGAGAGTT 3′
**Oct4**	
F	5′ CACAACGAGAGGATTTTGAGGCTG 3′
R	5′ GCAGTGAAGTGAGGGCTCCCATAG 3′
**SOX2**	
F	5′ CACCCCTGGCATGGCTCTTG 3′
R	5′ TGGAGTGGGAGGAAGAGGTAAC 3′

Table S2. Information on antibodies used in this study.

Antibody	WB	Specificity	Company
CD44v6(ab205719)	1:2000	Mouse polyclonal	Abcam
CD44(ab243894)	1:1000	Rabbit monoclonal	Abcam
CD24(ab290730)	1:1000	Rabbit monoclonal	Abcam
CD133 (ab19898)	1:1000	Rabbit polyclonal	Abcam
EpCAM(ab223582)	1:1000	Rabbit monoclonal	Abcam
β-actin（A5441）	1:10000	Mouse monoclonal	Sigma
